# Hypopigmented macules on the suprapubis and axillae

**DOI:** 10.1016/j.jdcr.2024.01.040

**Published:** 2024-03-15

**Authors:** Clint Christian T. Garbanzos, Madeleine G. Sowash, Michael A. Cardis, A. Yasmine Kirkorian

**Affiliations:** a Department of Pathology and Laboratory Medicine, MedStar/Georgetown University Hospital, Washington, District of Columbia; b Department of Dermatology, Washington Hospital Center, Washington, District of Columbia; c Division of Dermatology, Children’s National Hospital, Washington, District of Columbia

**Keywords:** axillae, clear cell papulosis, skin, suprapubic

## Case presentation

**Figure 1:**
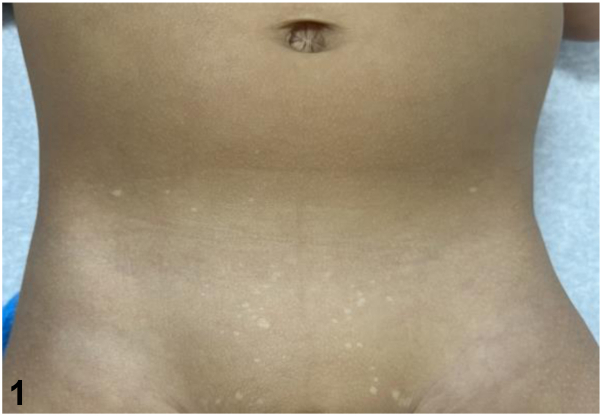

**Figure 2:**
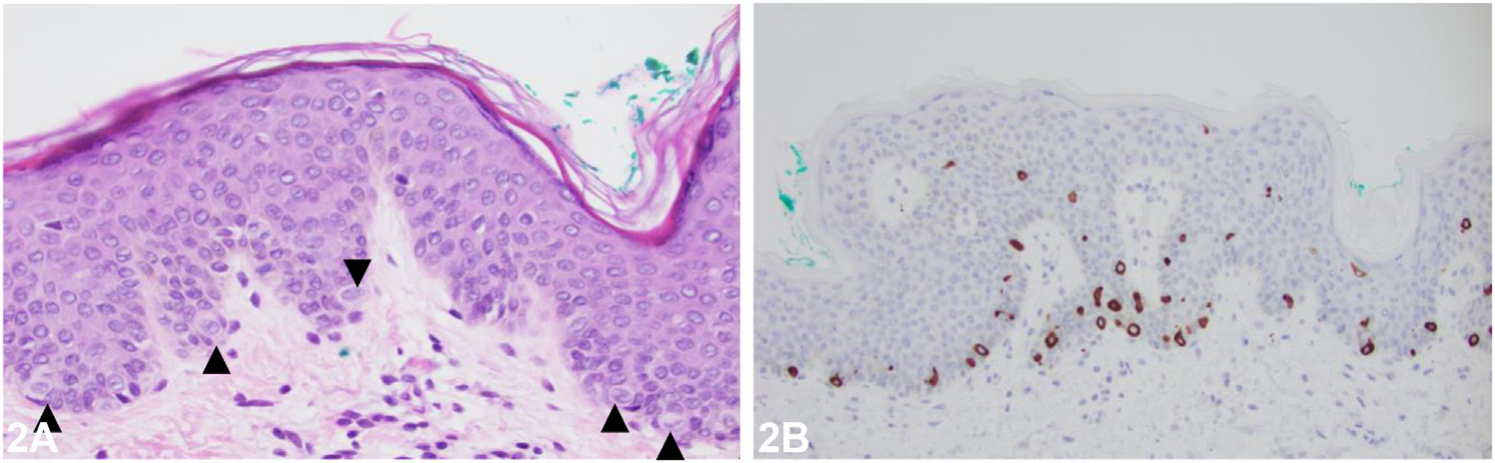

A 1-year-and-8-month-old healthy Hispanic male is seen at the clinic due to a 1-year history of multiple stable, hypopigmented and asymptomatic macules, and papules on his suprapubic area and axillae. He had no prior treatment and no relevant past medical history. On physical examination, the lesions were found to be well-demarcated, 2–4 mm, hypopigmented macules on the suprapubic skin, lower abdomen (Fig 1), and axillae. The histopathological examination of one of the lesion is shown in Fig 2, *A* and *B*, demonstrating the presence of several CK7-positive large clear cells (designated by an arrowhead) in the epidermis (i.e. Toker cells).

**Question 1: What is the most likely diagnosis?**

A. Tinea versicolor
B. Confetti macules of tuberous sclerosis
C. Primary extramammary Paget disease
D. Clear cell papulosis
E. Verruca plana

**Answers:**

**A.** Tinea versicolor – Incorrect. Tinea versicolor is caused by *Malassezia* species and presents with hypo- or hyperpigmented macules with fine inducible scale, most commonly in young adults. Histology characteristically shows yeast and hyphal forms within the stratum corneum associated with orthokeratotic hyperkeratosis and would lack numerous large epithelioid cells with clear cytoplasm and vesicular nuclei (ie. Toker cells) within the epidermis.
**B.** Confetti macules of tuberous sclerosis – Incorrect. Tuberous sclerosis can present with hypopigmented macules, but this not an isolated finding. Histology characteristically shows normal skin with significantly reduced melanin and no abundance of Toker cells within the epidermis.
**C.** Primary extramammary Paget disease – Incorrect. This is a malignant skin disease speculated to arise from a hyperplastic focus of Toker cells.^1^ It is a close histopathologic differential for clear cell papulosis, but their clinical presentations are distinct. Primary extramammary Paget disease typically affects older individuals and presents with an eczematous rash in apocrine-gland bearing areas.^1^
**D.** Clear cell papulosis – Correct. Clear cell papulosis is a rare skin condition that occurs in otherwise healthy patients ranging from birth to less than 6 years old, typically in those with Asian or Hispanic ancestry.^2^ It presents as multiple, nonprogressing, asymptomatic, hypopigmented macules, and flat-topped papules that occur in apocrine-gland bearing areas; typically, along the milk lines including the suprapubic area and axillae.^1^^,^^2^ Histology reveals multiple large epithelioid cells with clear cytoplasm and vesicular nuclei (ie. Toker cells) within an otherwise normal-appearing epidermis.^3^
**E.** Verruca plana – Incorrect. Verruca plana presents with flat-topped papules due to human papillomavirus infection. Histology characteristically shows orthokeratosis, mild acanthosis, hypergranulosis, and vacuolated keratinocytes in the upper dermis without the presence of multiple Toker cells.

**Question 2: Which of the following is TRUE of Toker cells?**

A. Malignant forms are found in Langerhans cell histiocytosis
B. Give rise to secondary extramammary Paget disease
C. Found at apocrine-bearing sites
D. Associated with cerbB-2 and Ki-67 positivity
E. Associated with viral infection

**Answers:**

**A.** Malignant forms are found in Langerhans cell histiocytosis – Incorrect. There is no documented evidence to imply an association between Toker cells and Langerhans cell histiocytosis.
**B.** Give rise to secondary extramammary Paget disease – Incorrect. Toker cells are believed to be the cells of origin of primary, not secondary, Paget disease.^1^ Secondary mammary and extramammary Paget disease are due to an underlying ductal carcinoma of the breast and carcinoma of the gastrointestinal or genitourinary tract, respectively.^4^
**C.** Found at apocrine-bearing sites – Correct. Toker cells are epithelial clear cells documented in nonlesional skin along the milk lines that bears apocrine glands such as the perineum, vulva, scrotum, suprapubic area, nipple and accessory breast tissues of the axillae.^1^^,^^2^ Normally, they are absent or sparsely present on routine hematoxylin and eosin-stained slides, typically in a suprabasal location. The cells are hypothesized to represent abortive mammary differentiation occurring either at embryonic or postnatal life. Hypopigmented lesions of clear cell papulosis are also seen in the apocrine-bearing sites; however, numerous Toker cells are easily identifiable among most basal keratinocytes upon microscopy.^3^
**D.** Associated with cerbB-2 and Ki-67 positivity – Incorrect. Although Toker cells and secondary mammary Paget cells are both CK7 and CEA positive, they can be differentiated by their cerbB-2, Ki-67 and ER staining patterns.^5^ Toker cells are typically cerbB-2 negative, Ki-67 negative and ER positive. Secondary mammary Paget cells are consistently cerbB-2 positive, Ki-67 positive and ER negative.^5^
**E.** Associated with viral infection – Incorrect. A koilocyte is associated with human papillomavirus infection of the basal keratinocyte. Toker cells are not directly associated with any viral infection.

**Question 3: Which of the following is the best next step in management?**

A. Perform an abdominopelvic computed tomography (CT) scan to rule out underlying malignancy
B. Apply topical salicylic acid under occlusion daily
C. Refer patient for genetic testing for mutations in TSC1 and TSC2
D. Apply topical ketoconazole daily for 2 weeks
E. Have the patient do yearly skin checks with the dermatologist

**Answers:**

**A.** Perform an abdominopelvic CT scan to rule out underlying malignancy – Incorrect. Secondary mammary and extramammary Paget disease are associated with underlying malignancy^1^ and warrant further radiologic investigation. Clear cell papulosis is not associated with an underlying malignancy and based on long-term follow up studies is unlikely to develop primary extramammary Paget disease.^3^
**B.** Apply topical salicylic acid under occlusion daily – Incorrect. Topical salicylic acid can be used to treat verruca plana. It is not a treatment that has been described for clear cell papulosis.
**C.** Refer patient for genetic testing for mutations in TSC1 and TSC2 – Incorrect. Mutations of the tumor suppressor genes, TSC1 and TSC2 are associated with tuberous sclerosis, a multisystem disease affecting the central nervous system, eyes, and kidneys, in addition to characteristic cutaneous findings. Clear cell papulosis is not associated with these mutations, and hence genetic testing is unwarranted.
**D.** Apply topical ketoconazole daily for 2 weeks – Incorrect. This is a topical antifungal used to treat tinea versicolor. Clear cell papulosis is not known to respond to antifungal treatment.
**E.** Have the patient do yearly skin checks with the dermatologist – Correct. Patients with a confirmed diagnosis of clear cell papulosis require no treatment since the skin lesions are asymptomatic. Many patients experience at least partial regression of the hypopigmented macules.^3^ Reassuring the parents of the benign nature of this condition in the majority of cases is prudent, however, yearly follow-up with a dermatologist is still warranted since there is a speculative link between Toker cells in clear cell papulosis and primary extramammary Paget disease.^1^

## Conflicts of interest

None disclosed.
